# Supplementary material for: Preclinical assessment of the efficacy and specificity of GD2-B7H3 SynNotch CAR-T in metastatic neuroblastoma
Source: Nat Commun. 2021 Jan 21;12:511. doi: 10.1038/s41467-020-20785-x (PMC7820416; doi:10.1038/s41467-020-20785-x)
Supplement: Supplementary file 1 — Supplementary Information [file 41467_2020_20785_MOESM1_ESM.pdf]

Preclinical Assessment of the Efficacy and Specificity of GD2-B7H3 SynNotch CAR-T in Metastatic Neuroblastoma

First Author: Babak Moghimi

Corresponding Author: Shahab Asgharzadeh

Supplementary information

Supplementary Figures 1-18

Supplementary Tables 1,2

Supplementary Figure 1.

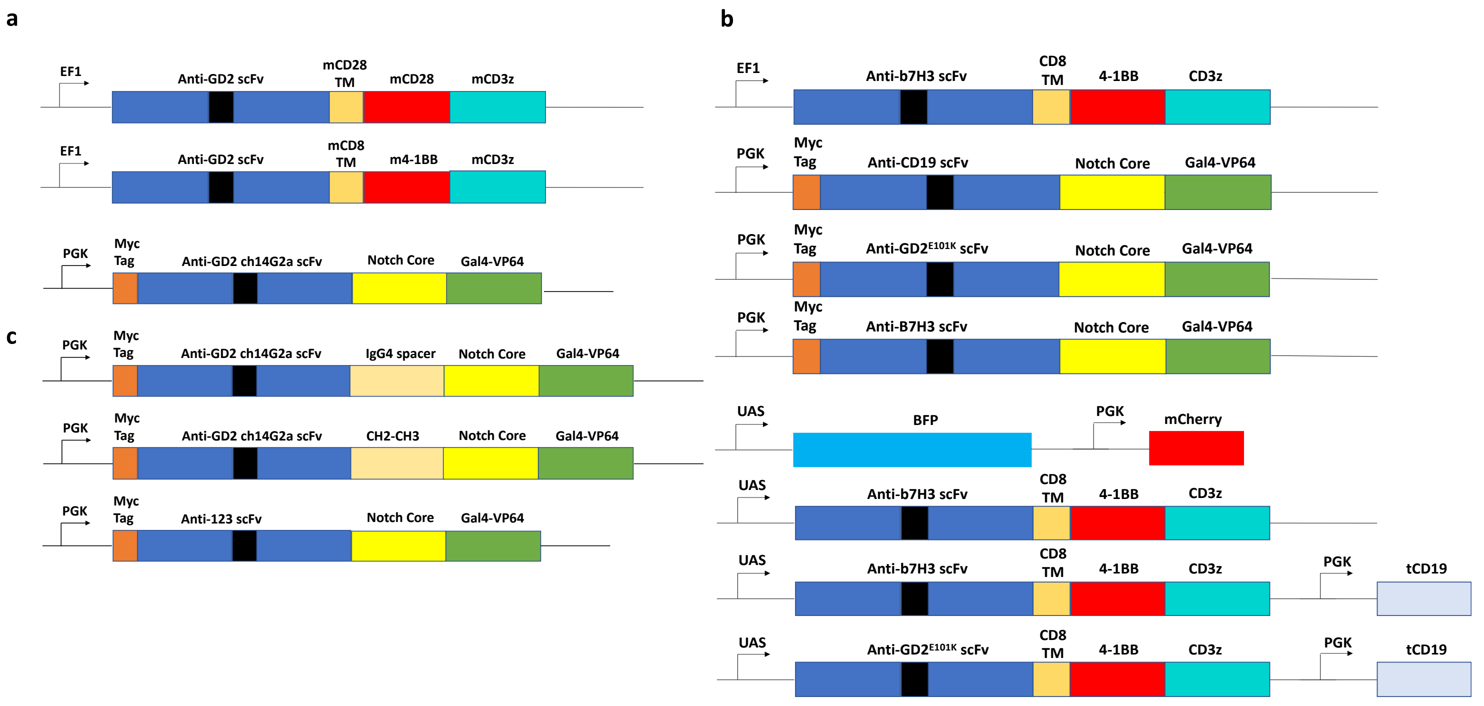

Supplementary Figure 1. Structure of CAR constructs. a Structures of murine GD2-28z and GD2-BBz CARs **b** Structures of B7H3-BBz, CD19-SynNotch, GD2^{E101K}-SynNotch, B7H3-SynNotch, Upstream activation sequence (UAS) responsive B7H3-BBz CAR, UAS responsive B7H3-BBz or GD2^{E101K}-BBz with PGK controlled truncated human CD19, UAS responsive BFP and PGK controlled mCherry. **c** Structures of SynNotch receptors for GD2 scFv (wildtype – corresponding to GD2 Chimeric 14G2a scFv) with no linker, IgG4, and CH2-CH3 linker, and SynNotch receptor for CD123 scFv.

Supplementary Figure 2.

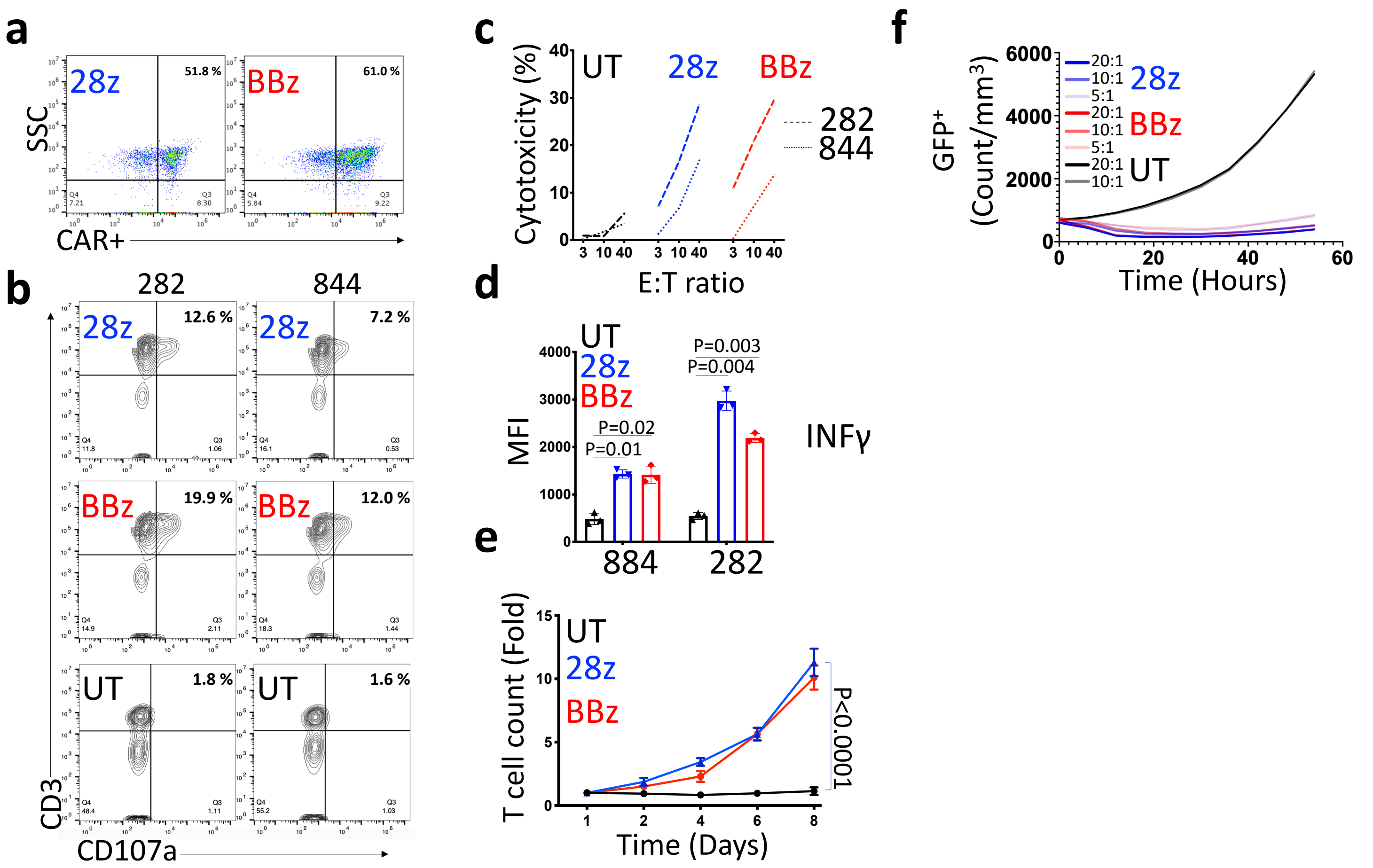

Supplementary Figure 2. **Murine GD2 CARs show anti-tumor efficacy *in vitro* and *in vivo*. a** Enumeration by flow cytometry of murine T cells transduced with retrovirus to express murine GD2-28z (28z) and GD2-BBz (BBz) construct. **b** Activation marker (CD107a) expression enumerated by flow cytometry in transduced T cells co-cultured with TH-MYCN murine NBL cell lines 282 and 844 for 4 hours. **c** Representation of cytotoxicity data from UT, GD2-28z, and GD2-BBz murine CAR-T cells co-cultured with murine TH-MYC NBL cell lines 282 and 844 for 12 hours at indicated E:T ratios. **d** Average IFNγ expression level in murine T cells by flow cytometry. **e** Average fold expansion of UT, GD2-28z, and GD2-BBz murine CAR-T cells co-cultured for 8 days with 282 murine NBL cells at 1:1 Effector:Target ratio. **f** Kinetics of average cytotoxicity of UT, murine GD2-28z and GD2-BBz CAR-T cells using live-cell imaging (enumeration of GFP^+ tumor cells) against 9464^{GD2+} murine NBL cells at indicated E:T ratios. Data shown are representative of three independent experiments (**a**-**e**). mean for minimum of 4 replicate (**f**). mean±SD (**d, e**). Two-tailed t-test (**d, e**). Source data are provided as a Source Data file.

Supplementary Figure 3.

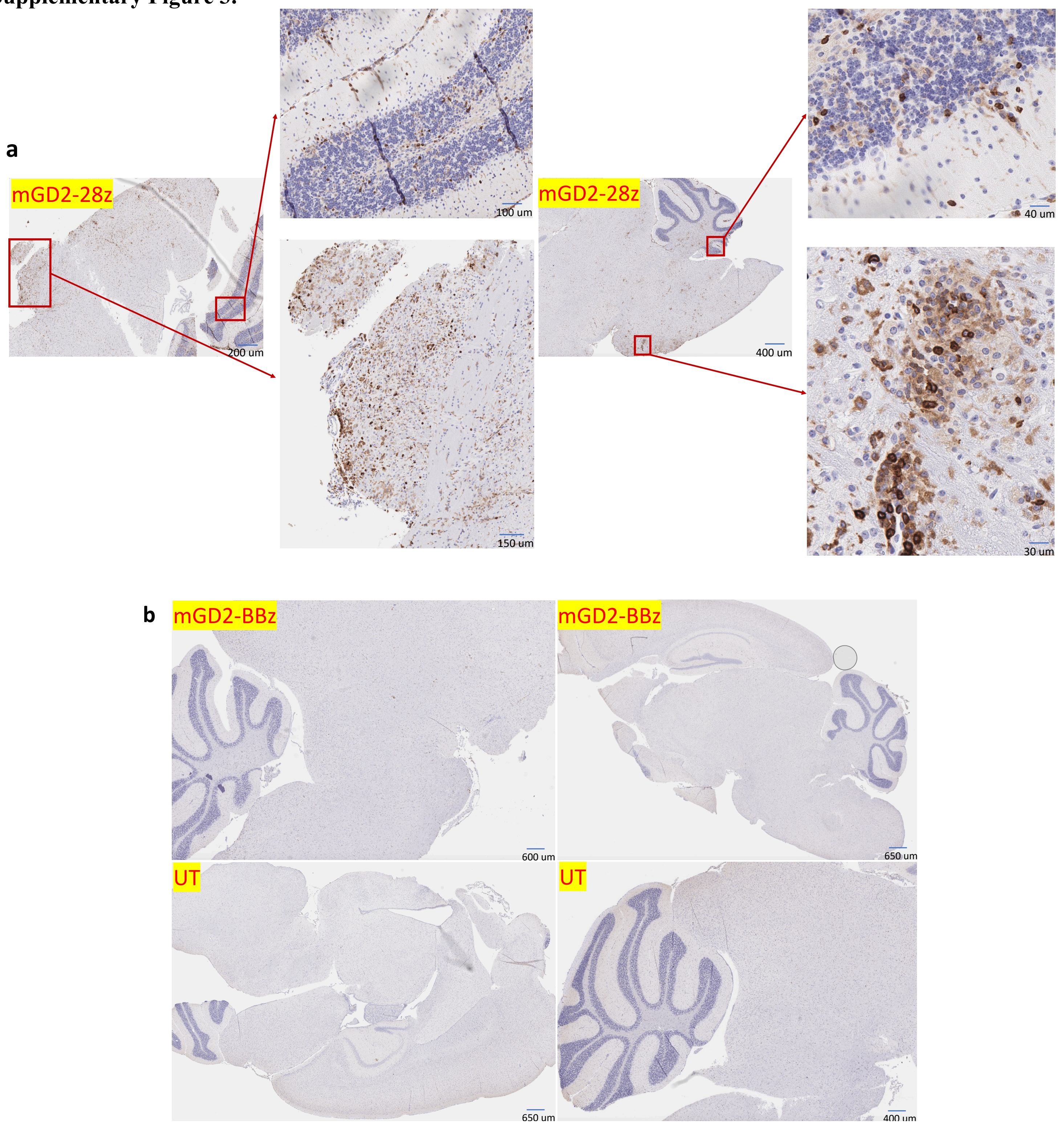

Supplementary Figure 3. **Immunohistochemical analysis of brain tissue from immunocompetent mice treated with murine GD2-28z.** Additional immunohistochemical analysis of CD3 (brown) in the brain of **a** animals treated with GD2-28z CAR-T cells, showing various regions of the central nervous system, and **b** animals treated with GD2-BBz CAR-T cells or UT cells, showing no infiltration in the central nervous system. Data shown are representative of the additional images from mice in Fig. 1c, no live animal was excluded.

Supplementary Figure 4

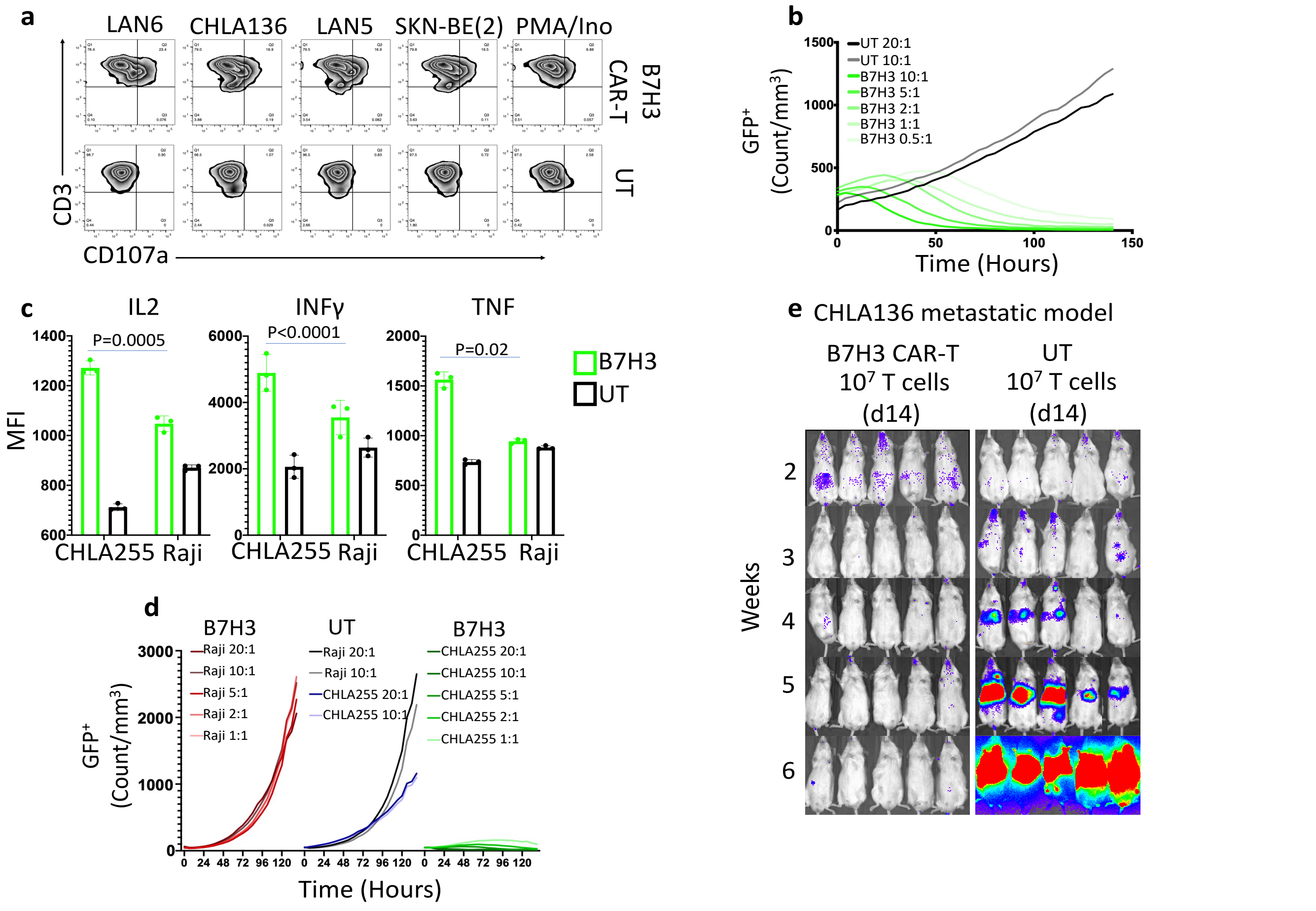

Supplementary Figure 4. Anti-tumor efficacy of B7H3 CAR-T cells against a range of NBL cell lines. a Enumeration by flow cytometry representing the expression of activation marker (CD107a) on transduced T cells ($CD3^+$) co-cultured with NBL cell lines for 4 hours. **b** Kinetics of cytotoxicity of UT and B7H3 CAR-T using live-cell imaging (enumeration of GFP^+ tumor cells) against B7H3+ CHLA255 cell line at indicated E:T ratios. **c** Summary of cytokine data as measured by flow cytometry. UT and B7H3 CAR-T co-cultured with $B7H3^+$ CHLA255 or $B7H3^-$ Raji cells at 1:1 E:T ratio for 24 hours. **d** Kinetics of cytotoxicity of UT and B7H3 CAR-T using live-cell imaging (enumeration of GFP^+ tumor cells) against B7H3+ CHLA255 and B7H3- Raji cell lines at indicated E:T ratios. **e** B7H3 CAR-T show *in* vivo efficacy against CHLA136 MYCN amplified NBL metastatic model. Bioluminescence images of CHLA136 tumor growth upon intravenous (i.v.) injection of NSG mice (1 x 10^6 cells/mouse) and treatment 14 days later with i.v. injection of 1x 10^7 B7H3 CAR-T cells or UT cells. Surviving mice were followed for a minimum of 100 days post tumor inoculation. Data shown are representative of three independent experiments(**a**,**c**). mean for minimum of 4 replicate (**b, d**). mean±SD (**c**). Two-tailed t-test (**c**). n=5 mice (B7H3, UT). Source data are provided as a Source Data file.

Supplementary Figure 5.

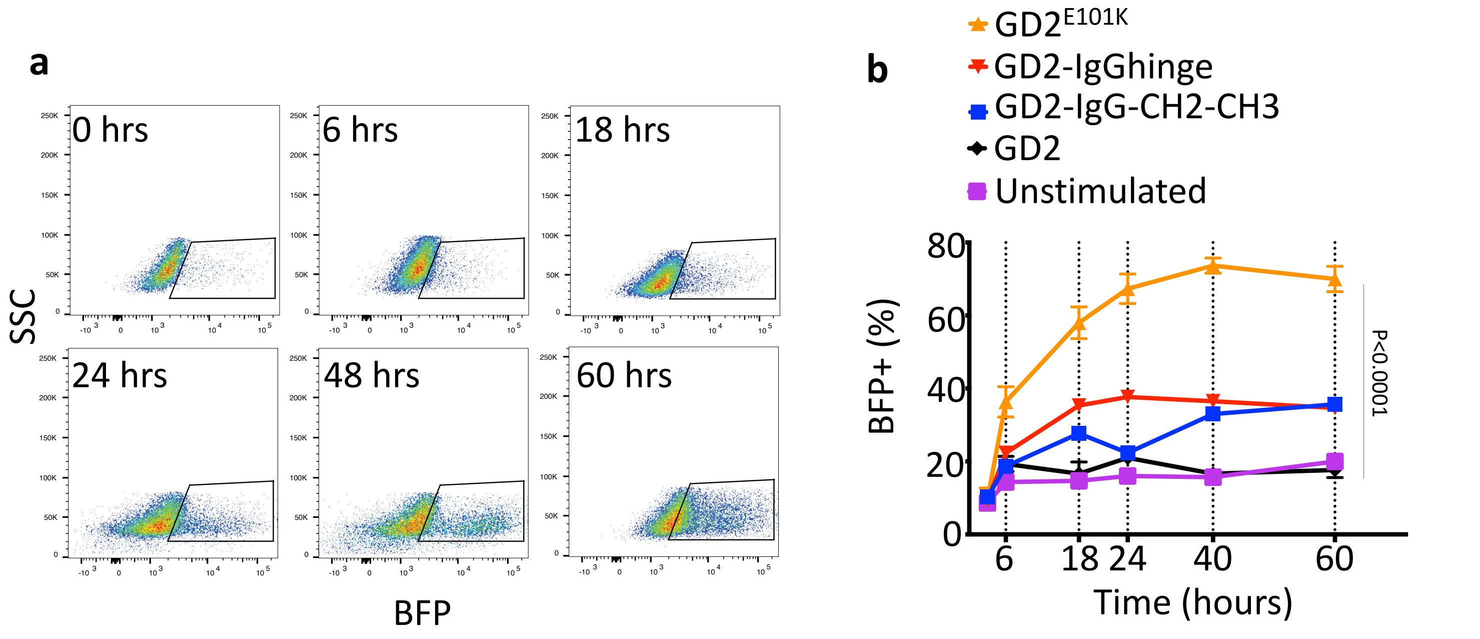

Supplementary Figure 5. **Kinetics of SynNotch activation with various gates. a** Representative dot plot of BFP expression by flow cytometry in GD2-BFP T cells co-cultured with NBL over a 3-day time course. **b** Time course kinetics of BFP expression by flow cytometry in $GD2^{E101K}$-BFP, $GD2^{WT}$-BFP, $GD2^{IgGhinge}$-BFP, and $GD2^{IgGCH2CH3}$-BFP T cells co-cultured with CHLA255 NBL cells for 3 days compared to unstimulated. Data shown are representative of three independent experiments (**a**, **b**). mean±SD (**b**). Two-tailed t-test (**b**)

Supplementary Figure 6.

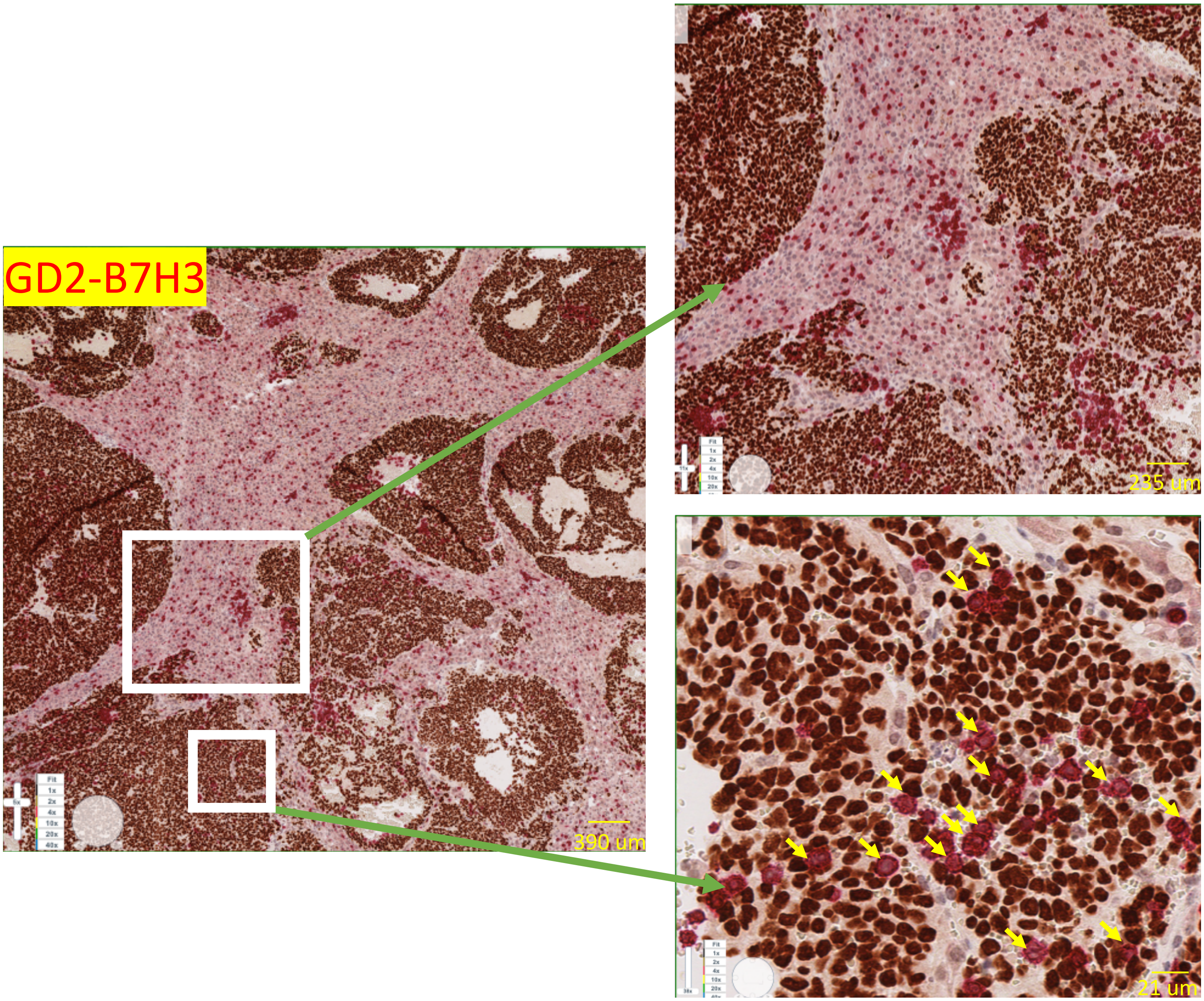

Supplementary Figure 6. **GD2-B7H3 T cells traffic and show evidence of cytotoxicity at metastatic NBL sites.** Representative images from immunohistochemical analysis of tissue obtained from the liver of mice injected i.v. with CHLA255 NBL cells (1 × 10^6 cells/mouse) followed by i.v. injection of GD2-B7H3 T cells on day 28 (high disease burden). Tissues were obtained 7 days post T-cell injections and stained for NBL specific marker Phox2b (brown) and human CD3 (red). Despite mice not surviving at this stage with high disease burden, gated T cell infiltration and minimal tumor reduction were identified. Yellow arrows point to T cells. Data shown are representative of three mice.

Supplementary Figure 7.

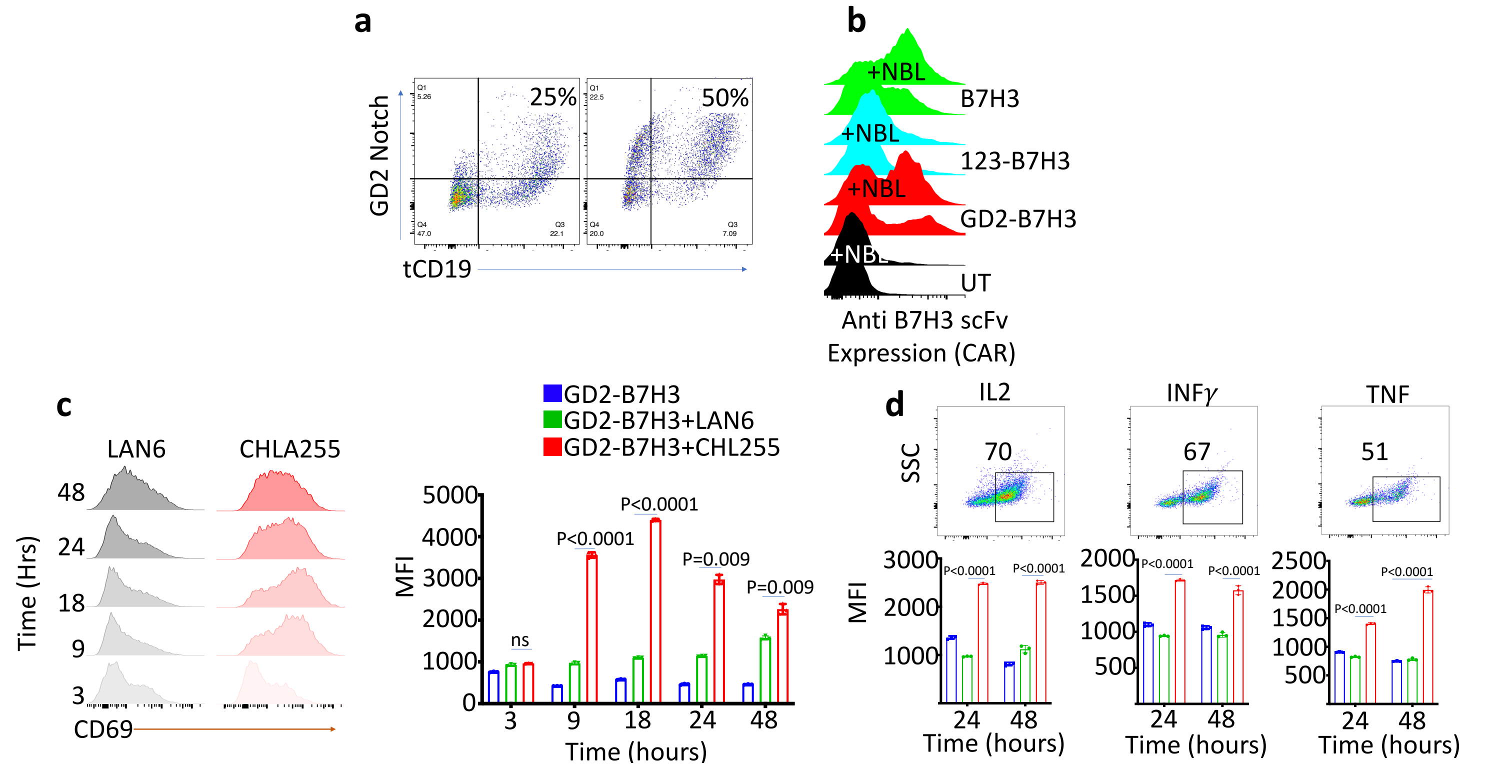

Supplementary Figure 7. **Enriched GD2-B7H3 T cells show *in vitro* specificity and efficacy**. **a** Representative dot plot of CD19 and GD2 SynNotch receptor expression in GD2-B7H3 T cells pre- and post-enrichment. **b** Histogram of B7H3 CAR expression as measured by flow cytometry analysis of conjugated B7H3 peptide binding on UT, B7H3 CAR-T, enriched GD2-B7H3, and CD123-B7H3 T cells with and without co-culturing CHLA255 NBL cells for 48 hours. **c** (Left) Representative histograms of early T cell activation marker (CD69) as measured by flow cytometry in enriched GD2-B7H3 T cells co-cultured with $GD2^{+}$ CHLA255 or $GD2^{-}$ LAN6 NBL cells at various time points as indicated. (Right) Summary of CD69 expression data measured by flow cytometry including unstimulated GD2-B7H3 T cells. **d** (Top) representative dot plots of intracellular cytokines IL2, IFNγ, and TNF in enriched GD2-B7H3 T cells co-cultured with CHLA255 NBL cells for 48 hours. (Bottom) Summary of cytokine expression as measured by flow cytometry for enriched GD2-B7H3 T cells co-cultured with $GD2^{+}$ CHLA255 or $GD2^{-}$ LAN6 NBL cells and unstimulated at 24 and 48 hours. Data shown are representative of three independent experiments (**a-d**). mean±SD (**c,d**). Two-tailed t-test (**c, d**). Source data are provided as a Source Data file

Supplementary Figure 8.

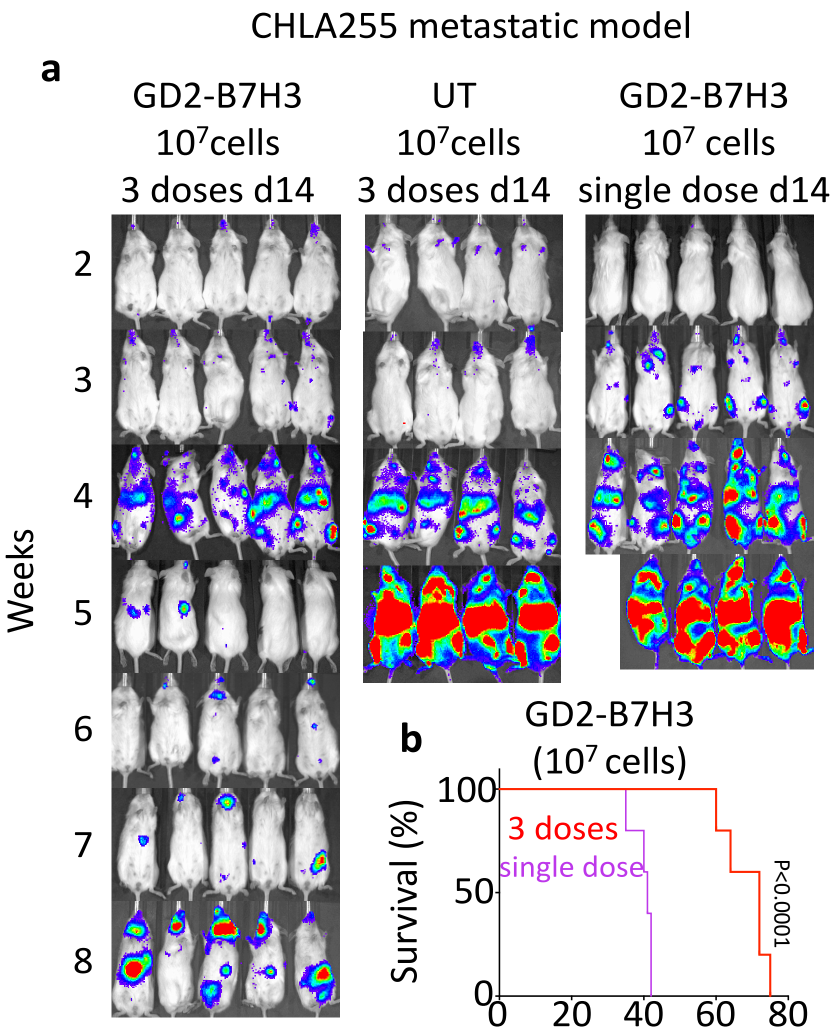

Supplementary Figure 8. **a GD2-B7H3 T cell reinfusion improves efficacy with no toxicity. a** Bioluminescence images of tumor growth in NSG mice after i.v. injection of CHLA255 NBL cells (1 x 10^6 cells/mouse) and treatment 14 days later with three doses of i.v. injection of UT or enriched GD2-B7H3 T cells (1X 10^7 cells/mouse/injection) versus one-time i.v. injection of GD2-B7H3 T cells. **b** Kaplan–Meier survival graph representing mice received 3 doses versus a single-dose i.v. injection of 1X 10^7 of GD2-B7H3 T cells on day 14 post tumor inoculation. n=5 mice (B7H3, GD2-B7H3 X3 dose) and n=4 (UT), no experimental mice excluded. Gehan-Breslow-Wilcoxon test (**b**).

Supplementary Figure 9.

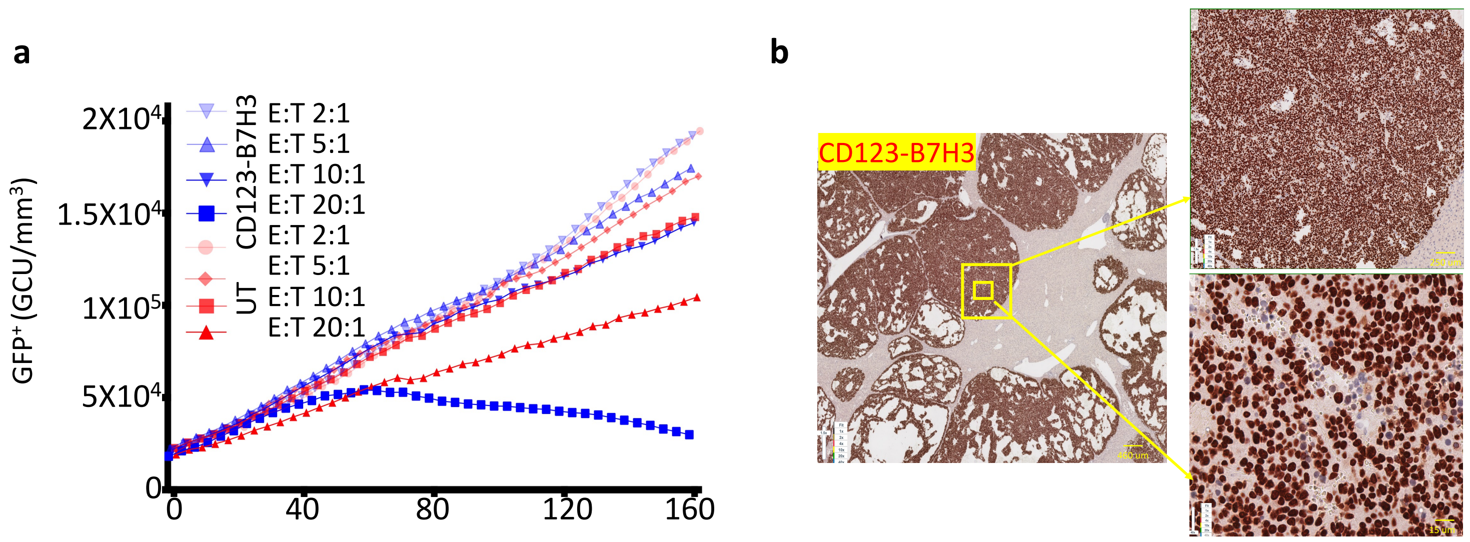

Supplementary Figure 9. **Leaky expression of B7H3 CAR in the gated system in functionally negligible**. **a** Kinetics of cytotoxicity of UT and CD123-B7H3 T using live-cell imaging (enumeration of GFP+ tumor cells) against CHLA255 NBL cells at indicated E:T ratios (minimum of 4 replicates). **b** Representative 1.5 X (Left), 10 X (Right upper), and 40 X (Right lower) images from immunohistochemical analysis of tissue obtained from the liver of mice injected i.v. with CHLA255 NBL cells (1×10^6 cells/mouse) followed by i.v. injection of $1X10^7$ CD123-B7H3 T cells per mouse on day 28 (high disease burden). Tissues were obtained 7 days post T-cell injections and stained for NBL specific marker Phox2b (brown) and human CD3 (red). CD123-B7H3 control gated T cell did not traffic to the tumor site. Mean for minimum of 4 replicate (**a**). n=2 mice (CD123-B7H3). source data are provided as a Source Data file.

Supplementary Figure 10.

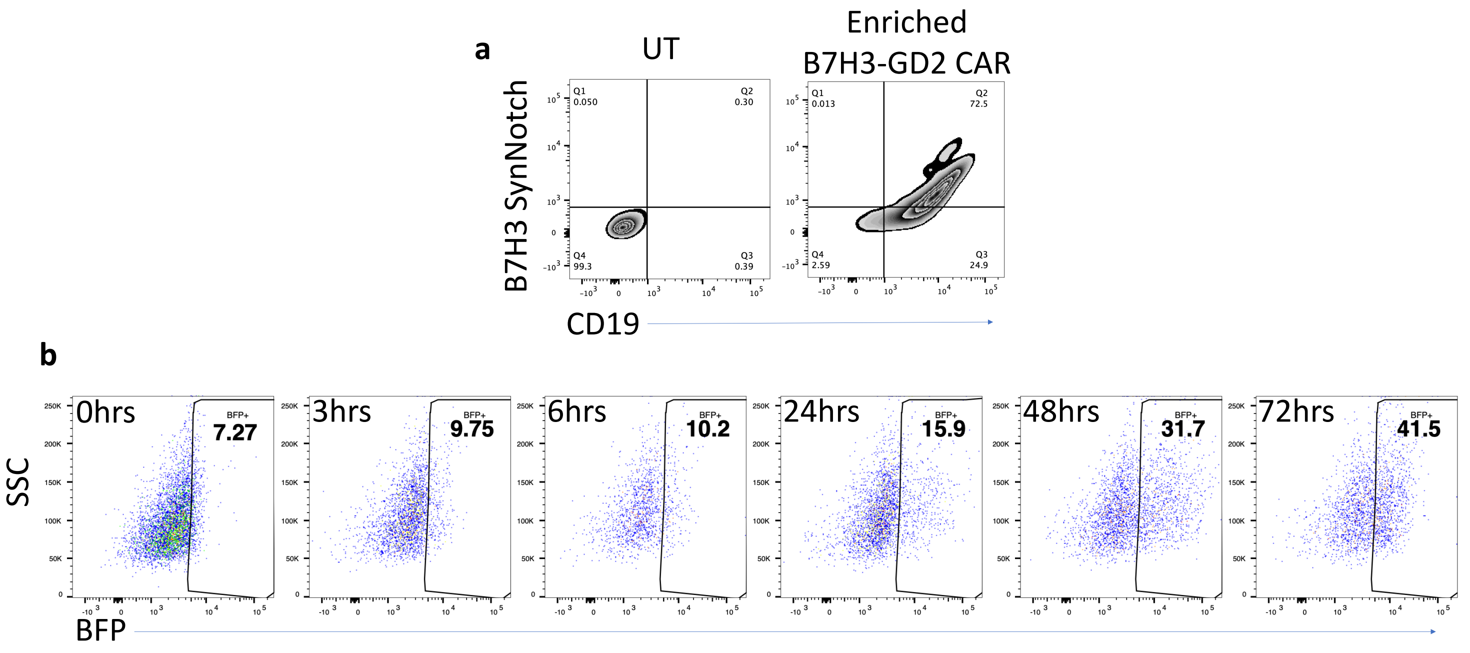

Supplementary Figure 10. **Enriched B7H3-GD2 system is functional in vitro**. **a** Contour plot representing the expression of SynNotch B7H3 gate and truncated CD19 by flow cytometry of untransduced (UT) and B7H3-GD2 transduced CAR T cells. **b** Representative dot plots of BFP expression measured by flow cytometry at indicated time points in B7H3-BFP T cells co-cultured with CHLA 255 NBL cells over a 3-day time course. Data shown are representative of three independent experiments (**a, b**).

Supplementary Figure 11.

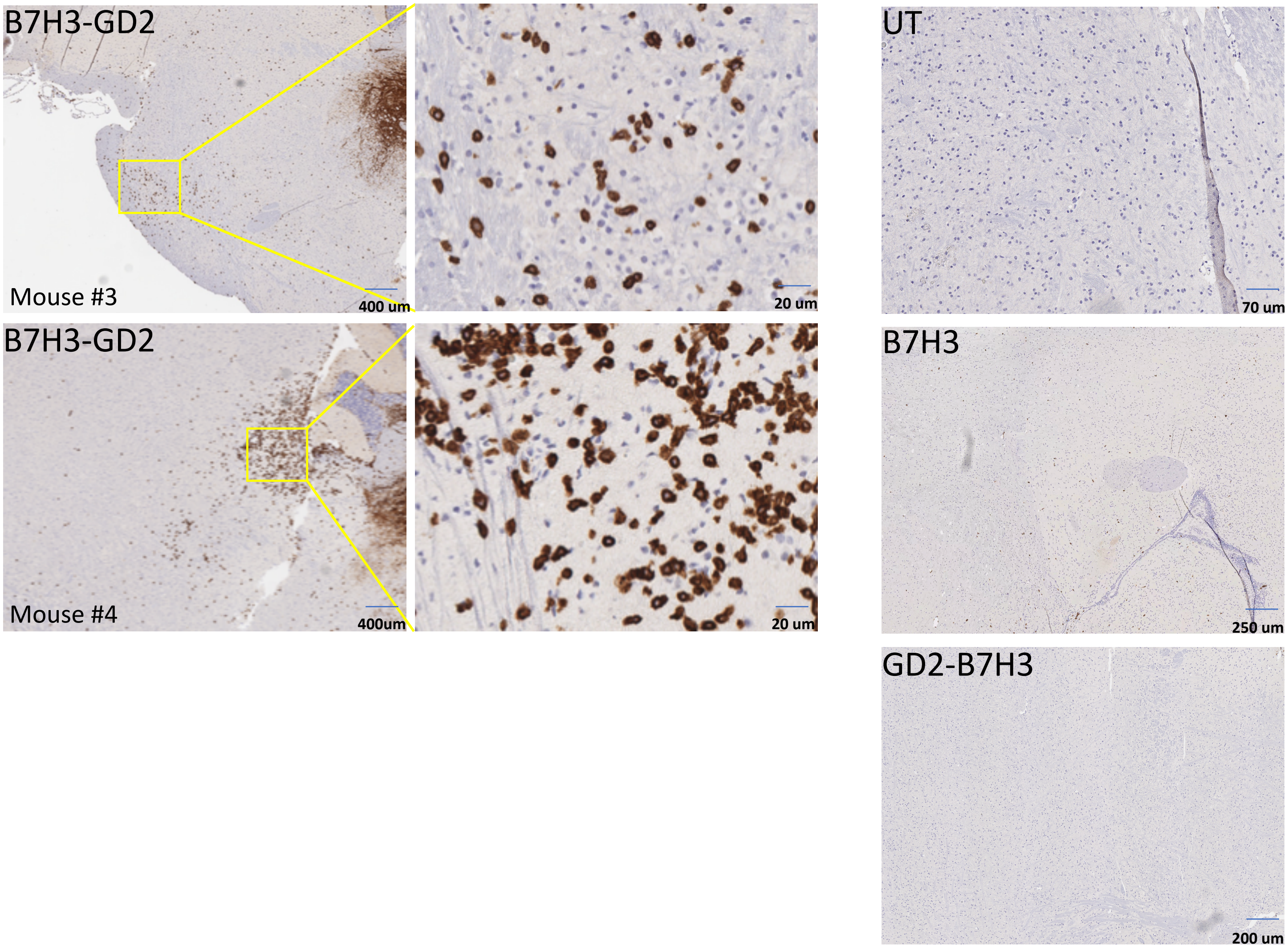

Supplementary Figure 11. Additional immunohistochemical analysis of CD3 expression (brown staining) in the brain of mice treated with B7H3-GD2 T cells, UT, B7H3, and GD2-B7H3 CAR-T cells. Data shown are representative of the additional images from mice in Fig. 7e, no live animal was excluded.

Supplementary Figure 12.

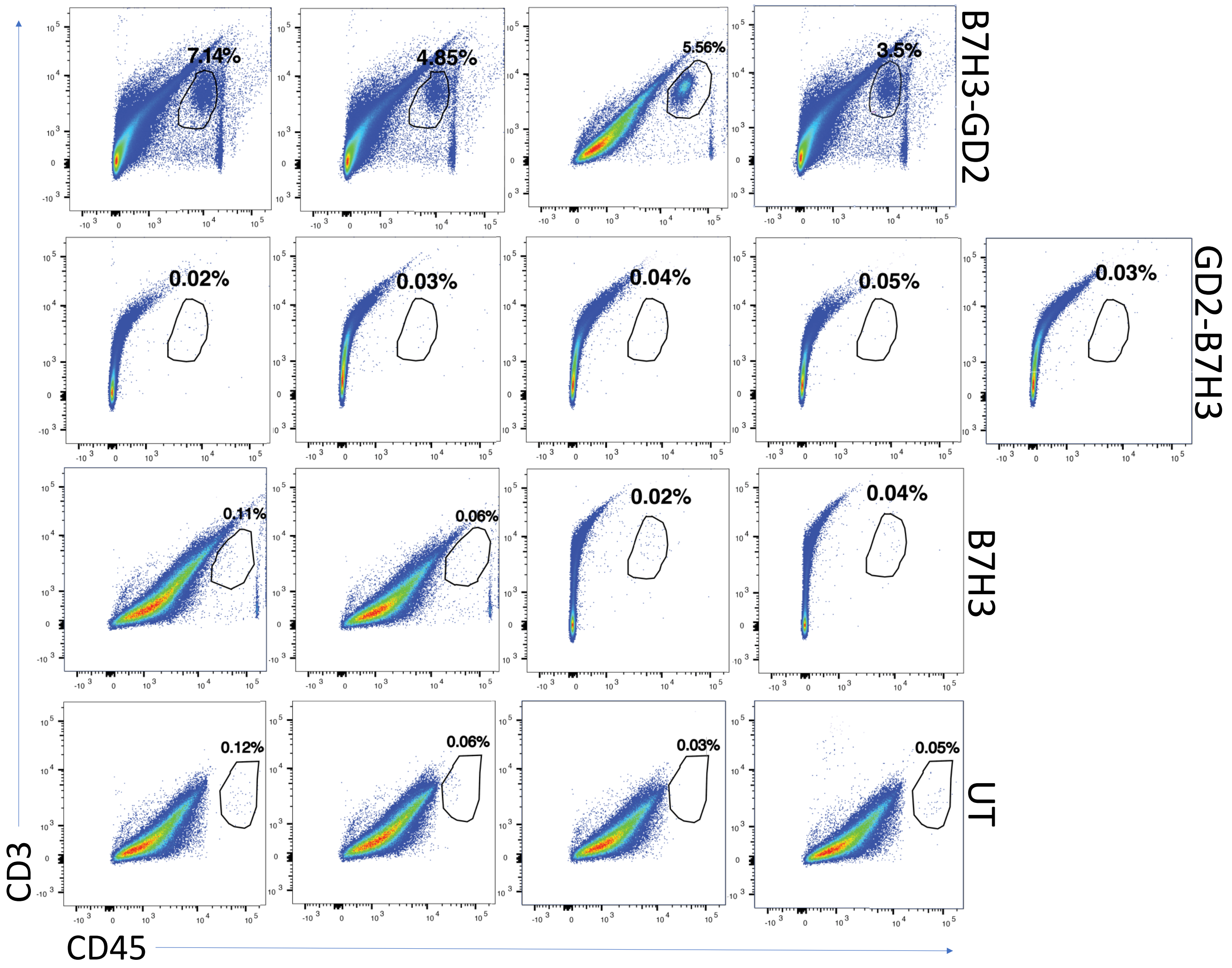

Supplementary Figure 12. Additional flow cytometry plots for CD45 and CD3 expression in single cells dissociated from brain tissue of animals treated with B7H3-GD2, GD2-B7H3, B7H3 CAR-T cells, and untransduced (UT) cells. no animal was excluded.

Supplementary Figure 13.

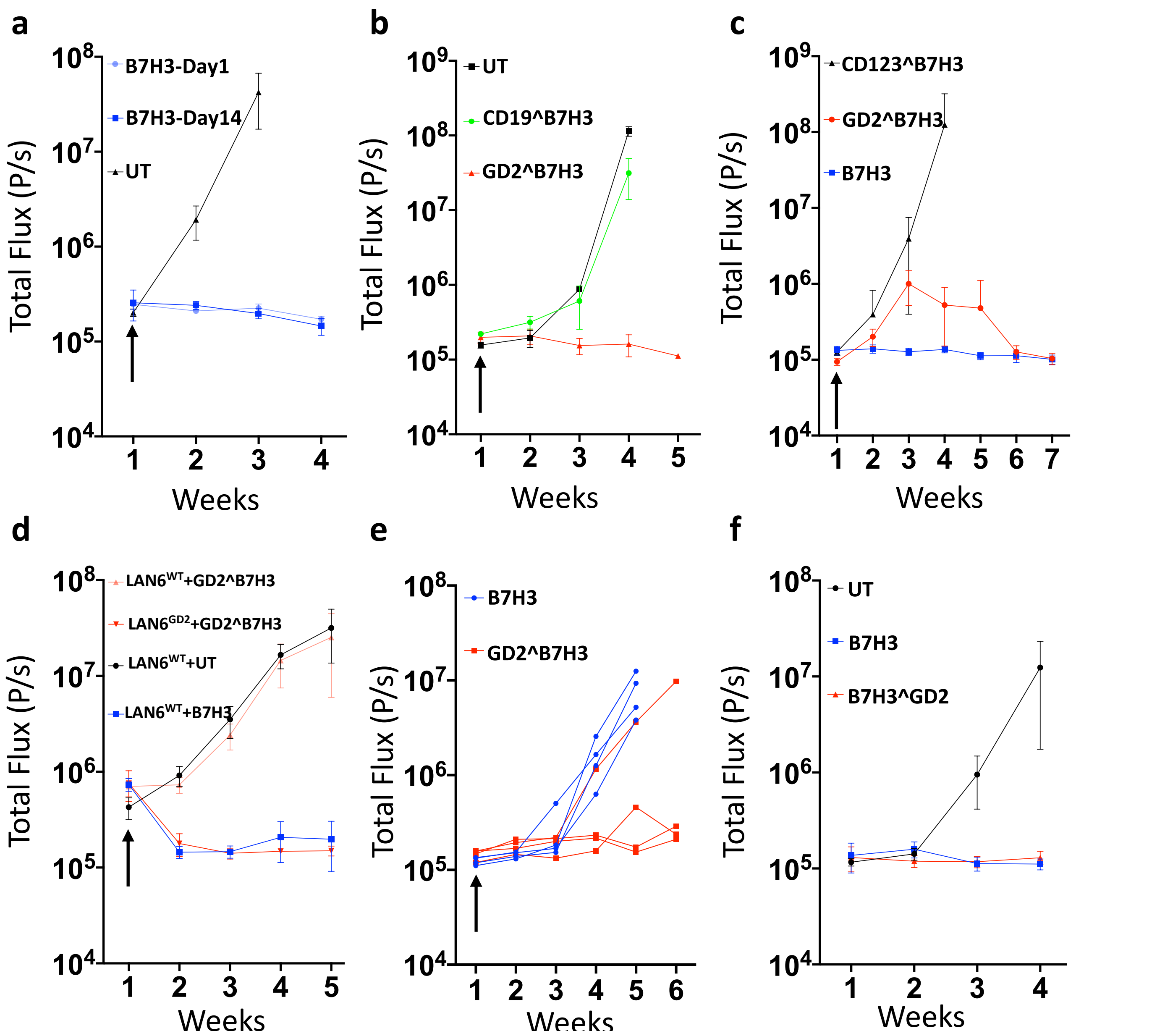

Supplementary Figure 13. Summary data representation of bioluminescence intensity in the main figures. **a-f** represent Figures 3g, 4j, 5g, 5f, 6g,7d.

Supplementary Figure 14.

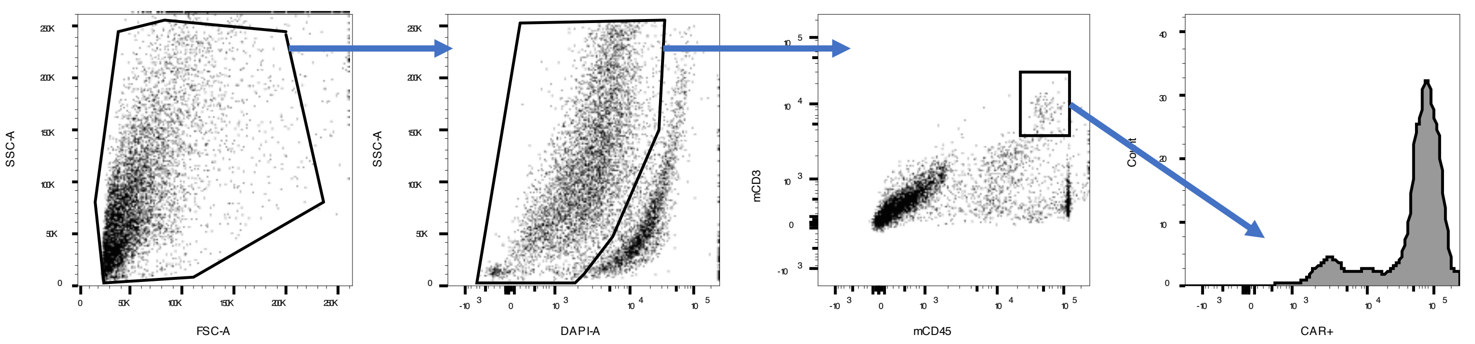

Supplementary Figure 14. Gating strategy to identify murine CAR-T cells in homogenized mice brain in Fig. 1(**b**). total live cells by DAPI were gated for mCD3 and mCD45. Fab^{+} CAR T cells were detected among double positive mouse T cells.

Supplementary Figure 15.

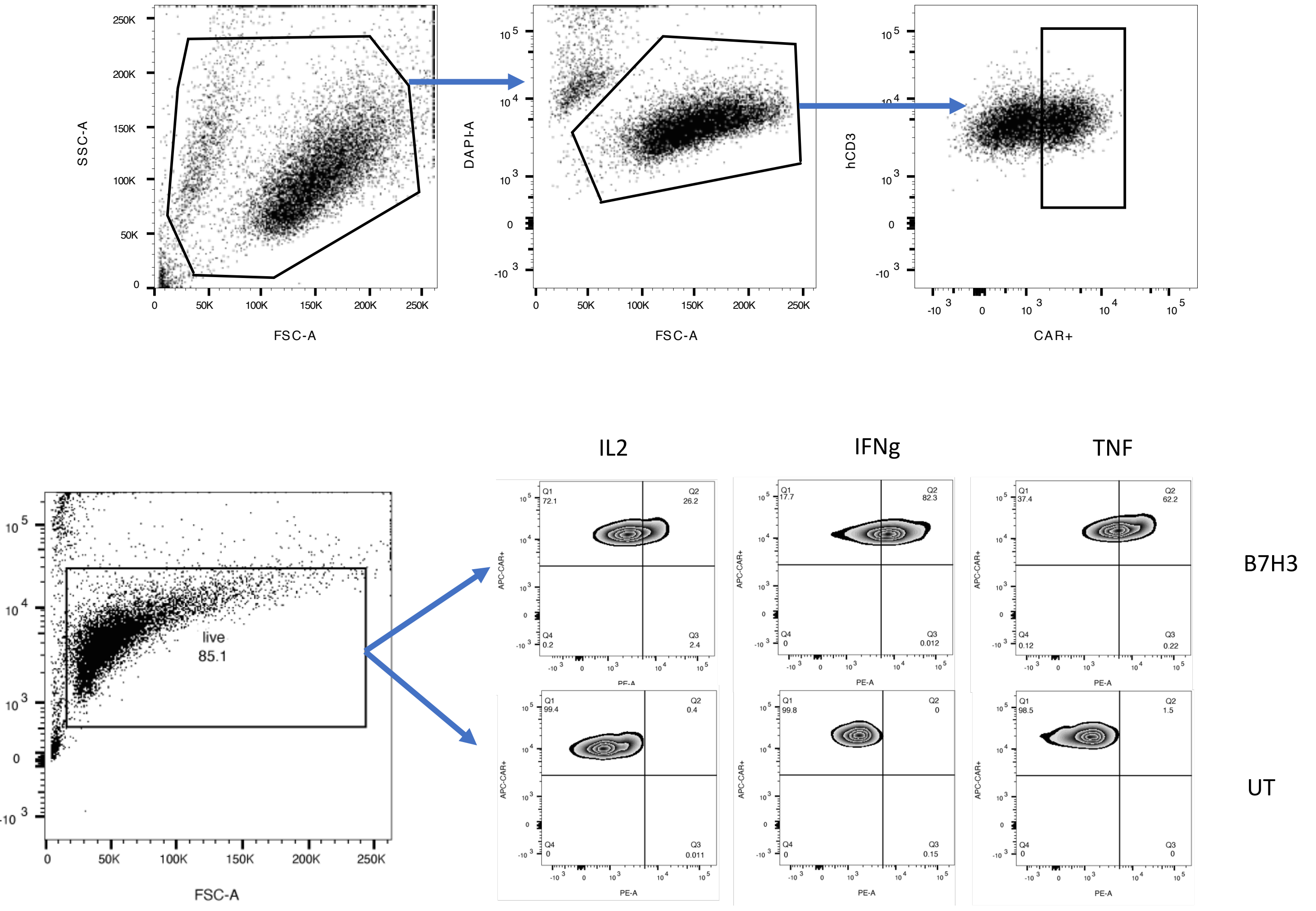

Supplementary Figure 15. Gating strategy to identify human CAR-T cells in Fig. 3 (**b,c**). **a** Total live cell by DAPI were gated for hCD3 and fab$^+$ CAR T cells. **b** Total live cells by fixable zombie uv were gated for APC-fab$^+$ CAR surface marker and intracellular cytokine in PE channel.

Supplementary Figure 16.

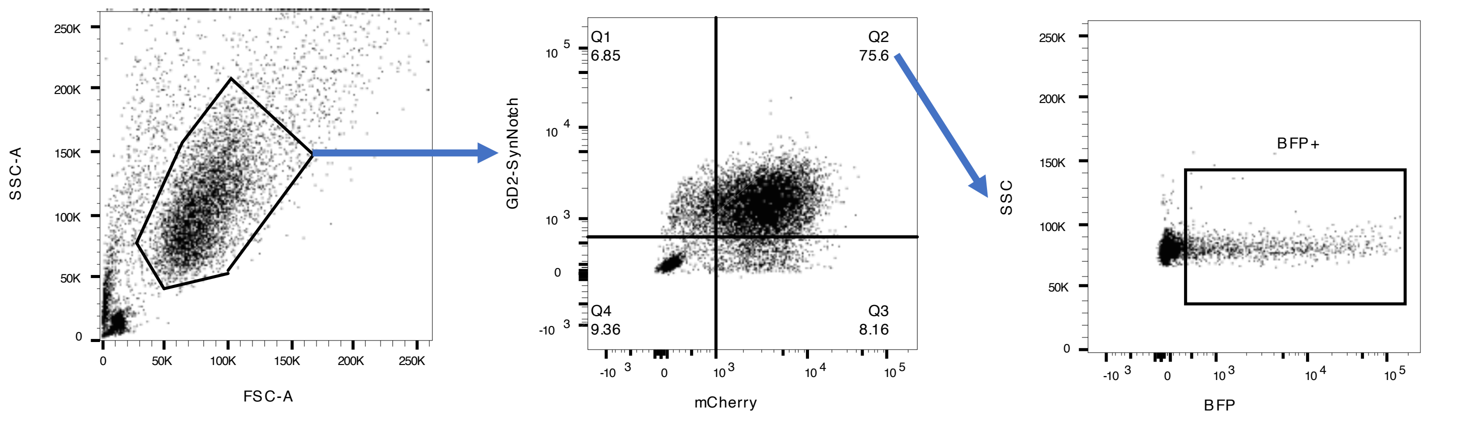

Supplementary Figure 16. Gating strategy to identify BFP expression in gated CAR-T cells**. a** Total cells were gated for SynNotch and mcherry expression (respond construct). Followed by BFP expression in double positive gate.

Supplementary Figure 17.

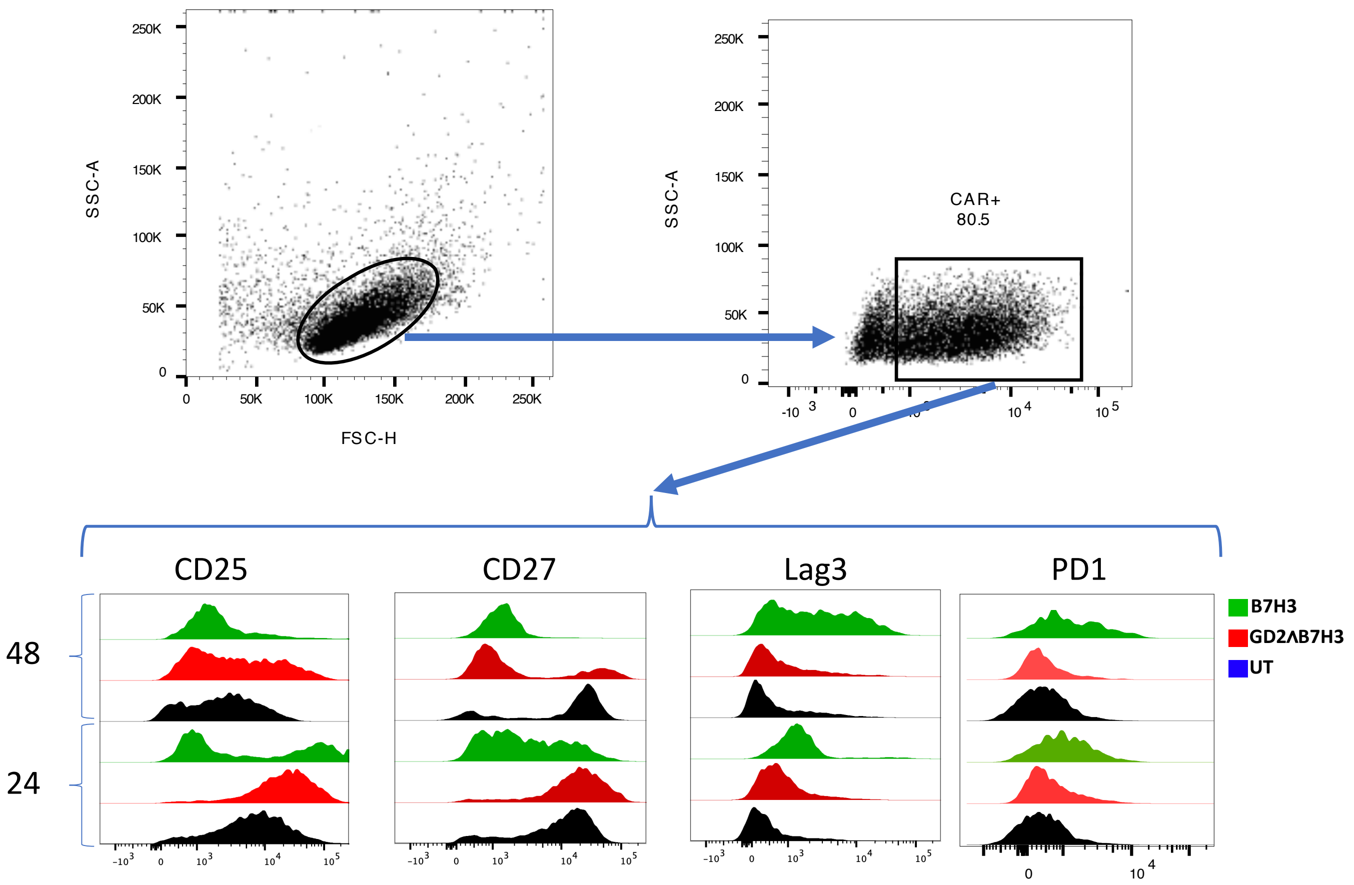

Supplementary Figure 17. Gating strategy to identify expression if the exhaustion marker in Fig. 5 (**a,b,f**) **.** Total cells were gated for CAR expression followed by individual expression of the markers measured in histogram.

Supplementary Figure 18.

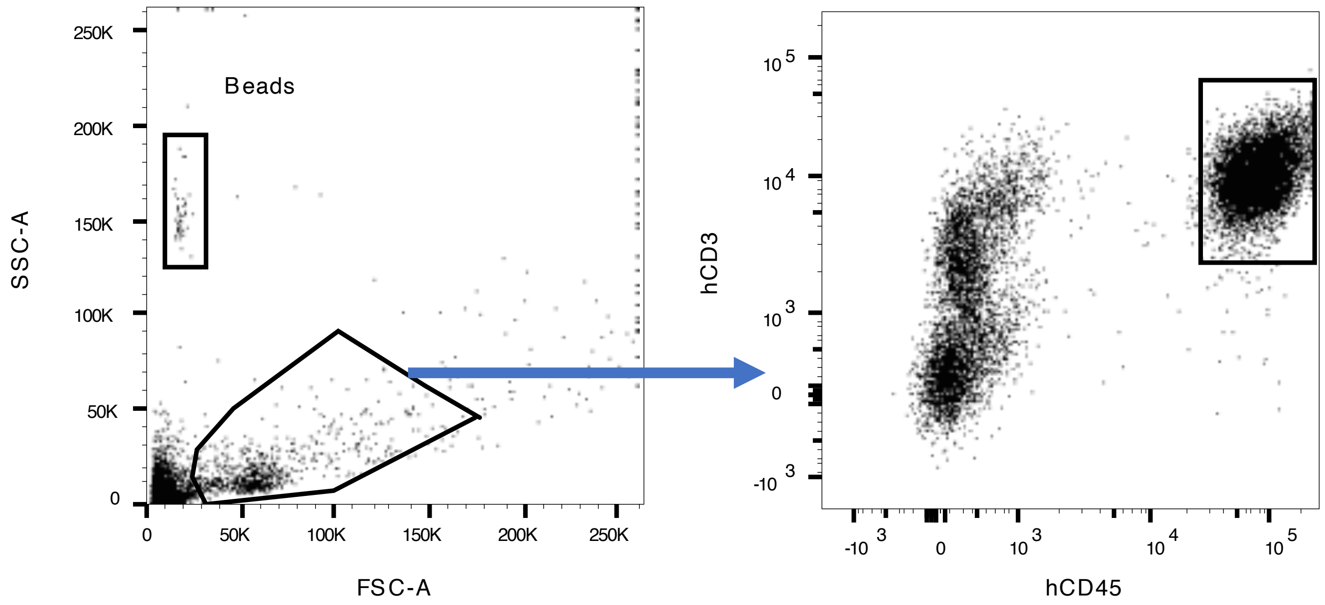

Supplementary Figure 18. Gating strategy to determine the human T cell count in mouse blood Fig. 5(**h**), 6(**h**) and 7(**f**). Total cells were gated for human CD3 and CD45 expression. Absolute T cell count/mm^3 was measured as T cell count/Beads *200.

Supplementary Table 1. List of reagents and antibodies

Reagent Type	Source/Sequence	Cat#	dilution
Antibodies			
AffiniPure F(ab')₂ Fragment Goat Anti-Mouse IgG, F(ab')₂ fragment sp	jackson immuno	cat#115-006-072	200
AffiniPure F(ab')₂ Fragment Goat Anti-Mouse IgG, F(ab')₂ fragment sp	jackson immuno	cat#115-006-072	200
AffiniPure F(ab')₂ Fragment Goat Anti-Mouse IgG, F(ab')₂ fragment sp	jackson immuno	cat#115-006-072	200
Pierce™ Recombinant Protein L, Biotinylated	Theromfisher	cat#29997	200
APC Streptavidin	Biolegend	cat#405243	200
PE anti-human IL-2	Biolegend	cat#500306	200
PE anti-human IFN-γ	Biolegend	cat#502508	200
PE anti-human TNF-α	Biolegend	cat#502908	200
FITC anti-human CD3	Biolegend	cat#317305	400
Brilliant Violet 421™ anti-human CD45	Biolegend	cat#368521	200
PE anti-human CD107a	Biolegend	cat#328607	200
PE anti-human CD279 (PD-1)	Biolegend	cat#329905	200
PE anti-human CD223 (LAG-3)	Biolegend	cat#369305	200
PE anti-human CD25	Biolegend	cat#302605	200
PE anti-human CD27	Biolegend	cat#356405	200
PE anti-human CD19	Biolegend	cat#392505	200
PE anti-human CD69	Biolegend	cat#310905	200
PE anti-human Ganglioside GD2	Biolegend	cat#357303	200
PE anti-human CD276 (B7-H3)	Biolegend	cat#331605	200
anti human CD3e Monoclonal	eBioscience	cat#14-0031-82	500
anti human PHOX2B Polyclonal	eBioscience	cat#PA5-35044	500
FITC anti-mouse CD3ε	Biolegend	cat#100305	400
Brilliant Violet 421™ anti-mouse CD45	Biolegend	cat#103133	400
PE anti-mouse CD107a	Biolegend	cat#121611	200
PE anti-mouse INFg	Biolegend	cat#505807	200
CFSE Cell Division Tracker Kit	Biolegend	cat#423801	
Recombinant Human B7-H3 (4Ig)/B7-H3b Protein	R&D systems	cat#2318-B3-050	
Critical Commercial Assays			
EasySep Human naive T Cell Isolation Kit	Stem Cell Technologies	Cat# 17951	
RNeasy Micro kit	QIAGEN	Cat# 74004	
MojoSort™ Mouse anti-PE Nanobeads	biolegend	Cat# 480079	
MojoSort™ Mouse CD45 Nanobeads	biolegend	Cat# 480027	
PE / R-Phycoerythrin Conjugation Ki	abcam	cat#ab102918	
MycoAlert™ Mycoplasma Detection Kit	lonza	cat#LT07-31	
BD QuantibriteTM Beads	BD	cat#340495	
Seahorse XF Cell Mito Stress Test Kit	Agilent	cat#103015-100	
Mouse T-Activator CD3/28 Dynabeads	Thermo Fisher Scientific	Cat# 11452D	
Human T-Activator CD3/28 Dynabeads	Thermo Fisher Scientific	Cat# 11132D	
Retronectin	Takara	Cat# T202	
Cell lines			
CHLA255			
LAN1			
LAN6			
CLHA136			
CHLA51			
SK.N.BE			
SMS-SAN			
293T			
jurkat			
ECO-phoenix			
Raji			
NBL9464			
Experimental Models: Organisms/Strains			
Mouse: C57BL/6 (B6): C57BL/6J	The Jackson Laboratory stock # 000664	Cat# JAX:000664; RRID: IMSR_JAX:00066	
Mouse: NOD/SCID/ γc-/- (NSG): NOD.Cg-PrkdcscidIl2rgtm1Wjl/SzJ	The Jackson Laboratory stock # 005557	Cat# JAX:005557; RRID: IMSR_JAX:005557	

Supplementary Table 2. Protein sequences of the constructs

Peptides, and Recombinant Proteins	
CD8 leader	MALPVTALLLPLALLLHAARP
CD8 transmembrane	IYIWAPLAGTCGVLLLSLVITLYC
4-1BB	KRGRKKLLYIFKQPFMRPVQTTQEEDGCSCRFPEEEEGGCEL
CD3z	RVKFSRSADAPAYKQGQNQLYNELNLGRREEYDVLDKRRGRDPEMGGKPRRKNPQEGLYNELQKDKMAEAYSEIGMKGERRR<br>GKGHDGLYQGLSTATKDTYDALHMQALPPR
GD2 HC	EVKLQQSGPSLVEPGASVMISCKASGSSFTGYNMNWVRQNIGKSLEWIGAIDPYYGGTSYNQKFKGRATLTVDKSSSTAYMHLKS<br>LTSEDSAVYYCVSGMEYWGQGTSVTVSSAKTTPPSVY
GD2 HC E101K	EVKLQQSGPSLVEPGASVMISCKASGSSFTGYNMNWVRQNIGKSLEWIGAIDPYYGGTSYNQKFKGRATLTVDKSSSTAYMHLKS<br>LTSEDSAVYYCVSGMKYWGQGTSVTVSSAKTTPPSVY
GD2 LC	DILLTQTPLSLPVSLGDQASISCRSSQSLVHRNGNTYLHWYLQKPGQSPKLLIHKVSNRFSGVPDRFSGSGSGTDFTLKISRVEAEDL<br>GVYFCSQSTHVPPLTFGAGTKLELKRADAAPTVSIFP
anti CD123 HC	QIQLVQSGPELKKPGETVKISCKASGYIFTNYGMNWVKQAPGKSFKWMGWINTYTGESTYSADFKGRFAFSLETSASTAYLHINDL<br>KNEDTATYFCARSGGYDPMDYWGQGTSVTVSS
anti CD123 LC	DIVLTQSPASLAVSLGQRATISCRASESVDNYGNTFMHWYQQKPGQPPKLLIYRASNLESGIPARFSGSGSRTDFTLTINPVEADDV<br>ATYYCQQSNEDPPTFGAGTKLELK
anti B7H3 HC	DVQLVESGGGLVQPGGSRKLSCAASGFTFSSFGMHWVRQAPEKGLEWVAYISSDSSAIYYADTVKGRFTISRDNPKNTLFLQMTS<br>LRSEDTAMYYCGRGRENIYYGSRLDYWGQGTTLTVSS
anti B7H3 LC	DIAMTQSQKFMSTSVGDRVSVTCKASQNVDTNVAWYQQKPGQSPKALIYSASYRYSGVPDRFTGSGSGTDFTLTINNVQSEDLA<br>EYFCQQYNNYPFTFGSGTKLEIK
CH2CH3	PPSVYESKYGPPCPSCPAPEFEGGPSVFLFPPKPKDTLMISRTPEVTCVVVDVSQEDPEVQFNWYVDGVEVHNAKTKPREEQFQST<br>YRVVSVLTVLHQDWLNGKEYKCKVSNKGLPSSIEKTISKAKGQPREPQVYTLPPSQEEMTKNQVSLTCLVKGFYPSDIAVEWESNG<br>QPENNYKTTPPVLDSDGSFFLYSRLTVDKSRWQEGNVFSCSVMHEALHNHYTQKSLSLSLGKILDYS
IgG4 hindge	EPKSCDKTHTCP
tCD19	MPPPRLLFFLLFLTPMEVRPEEPLVVKVEEGDNAVLQCLKGTSDGPTQQLTWSRESPLKPFLKLSLGLPGLGIHMRPLAIWLFIFNV<br>SQQMGGFYLCQPGPPSEKAWQPGWTVNVEGSGELFRWNVSDLGGLGCGLKNRSSEGPSSPSGKLMSPKLYVWAKDRPEIWEG<br>EPPCLPPRDSLNQSLSQDLTMAPGSTLWLSCGVPPDSVSRGPLSWTHVHPKGPKSLLSLELKDDRPARDMWVMETGLLLPRATA<br>QDAGKYYCHRGNLTMSFHLEITARPVLWHWLLRTGGWKVSAVTLAYLIFCLCSLVGILHLQRALVLRRKRKRMTDPTRRFFKVTP<br>PPGSGPQNQYGNVLSLPTPTSGLGRAQRWAAGLGGTAPSYGNPSSDVQADGALGSRSPPGVGPEEEEGEGYEEPDSEEDSEFYE<br>NDSNLGQDQLSQDGSGYENPEDEPLGPEDEDSFSNAESYENEDEELTQPVARTMDFLSPHGSAWDPSREATSLGSQSYEDMRG<br>ILYAAPQLRSIRGQPGPNHEEDADSYENMDNPDGPDPAWGGGGRMGTWSTR*
mCD28	IEFMYPPPYLDNERSNGTIIHIKEKHLCHTQSSPKLFWALVVVAGVLFCYGLLVTVALCVIWTNSRRNRGGQSDYMNMTPRRPGL<br>TRKPYQPYAPARDFAAYRP
mCD3	RAKFSRSAETAANLQDPNQLFNELNLGRREEFDVLEKKRARDPEMGGKQQRRRNPQEGVYNALQKDKMAEAYSEIGTKGERRR<br>GKGHDGLFQGLSTATKDTFDALHMQTLAPR
mCD8	SSVVPVLQKVNSTTTKPVLRTPSPVHPTGTSQPQRPEDCRPRGSVKGTGLDFACDIYIWAPLAGICVALLLSLIITLI
m4-1BB	SVLKWIRKKFPHIFKQPFKKTTGAAQEEDACSCRCPQEEEGGGGGYEL
GD2-T2A-GD3 synthase	MEAGRPLGQRPIAALLLRFLGSEAGKGWVRGRAQGRAQGRGGRPKVLRRPGILHASKAHVCRAVLLFLISGPFEFSRVKWSKAG<br>GQWIMSPCGRARRQTSRGAMAVLAWKFPRTRLPMGASALCVVVLCWLYIFPVYRLPNEKEIVQGVLQQGTAWRRNQTAARAF<br>RKQMEDCCDPAHLFAMTKMNSPMGKSMWYDGEFLYSFTIDNSTYSLFPQATPFQLPLKKCAVVGNGGILKKSGCGRQIDEANF<br>VMRCNLPPLSSEYTKDVGSKSQLVTANPSIIRQRFQNLLWSRKTFVDNMKIYNHSYIYMPAFSMKTGTEPSLRVYYTLSDVGANQ<br>TVLFANPNFLRSIGKFWKSRGIHAKRLSTGLFLVSAALGLCEEVAIYGFWPFSVNMHEQPISHHYYDNVLPFSGFHAMPEEFLQLW<br>YLHKIGALRMQLDPCEDTSLQPTSGSGEGRGSLLTCGDVEENPGPMWLGRRALCALVLLLACASLGLLYASTRDAPGLRLPLAPW<br>APPQSPRRPELPDLAPEPRYAHIPVRIKEQVVGLLAWNNCSCESSGGGLPLPFQKQVRAIDLTKAFDPAELRAASATREQEFQAFLS<br>RSQSPADQLLIAPANSPLQYPLQGVEVQPLRSILVPGLSLQAASGQEVYQVNLTASLGTWDVAGEVTGVTLTGEGQADLTLVSPG<br>LDQLNRQLQLVTYSSRSYQTNTADTVRFSTEGHEAAFTIRIRHPPNPRLYPPGSLPQGAQYNISALVTIATKTFLRYDRLRALITSIRR<br>FYPTVTVVIADDSDKPERVSGPYVEHYLMPFGKGWFAGRNLAVSQVTTKYVLWVDDDFVFTARTRLERLVDVLERTPLDLVGGA<br>VREISGFATTYRQLLSVEPGAPGLGNCLRQRRGFHHELVGFPGCVVTDGVVNFFLARTDKVREVGFDPRLSRVAHLEFFLDGLGS<br>LRVGSCSDVVVDHASKLKLPWTSRDAGAETYARYRYPGSLDESQMAKHRLLFFKHRLQCMTSQ*
