# Supplementary material for: Preclinical assessment of the efficacy and specificity of GD2-B7H3 SynNotch CAR-T in metastatic neuroblastoma
Source: Nat Commun. 2021 Jan 21;12:511. doi: 10.1038/s41467-020-20785-x (PMC7820416; doi:10.1038/s41467-020-20785-x)
Supplement: Supplementary file 3 — Reporting Summary [file 41467_2020_20785_MOESM3_ESM.pdf]

nature research

Corresponding author(s): Shahab Asgharzadeh

Last updated by author(s): Nov 20, 2020

Reporting Summary

Nature Research wishes to improve the reproducibility of the work that we publish. This form provides structure for consistency and transparency in reporting. For further information on Nature Research policies, see our Editorial Policies and the Editorial Policy Checklist.

Statistics

For all statistical analyses, confirm that the following items are present in the figure legend, table legend, main text, or Methods section.

n/a	Confirmed	
☐	☒	The exact sample size (*n*) for each experimental group/condition, given as a discrete number and unit of measurement
☐	☒	A statement on whether measurements were taken from distinct samples or whether the same sample was measured repeatedly
☐	☒	The statistical test(s) used AND whether they are one- or two-sided<br>*Only common tests should be described solely by name; describe more complex techniques in the Methods section.*
☐	☒	A description of all covariates tested
☐	☒	A description of any assumptions or corrections, such as tests of normality and adjustment for multiple comparisons
☐	☒	A full description of the statistical parameters including central tendency (e.g. means) or other basic estimates (e.g. regression coefficient) AND variation (e.g. standard deviation) or associated estimates of uncertainty (e.g. confidence intervals)
☐	☒	For null hypothesis testing, the test statistic (e.g. *F*, *t*, *r*) with confidence intervals, effect sizes, degrees of freedom and *P* value noted<br>*Give P values as exact values whenever suitable.*
☒	☐	For Bayesian analysis, information on the choice of priors and Markov chain Monte Carlo settings
☒	☐	For hierarchical and complex designs, identification of the appropriate level for tests and full reporting of outcomes
☒	☐	Estimates of effect sizes (e.g. Cohen's *d*, Pearson's *r*), indicating how they were calculated

Our web collection on statistics for biologists contains articles on many of the points above.

Software and code

Policy information about availability of computer code

Data collection	IncuCyte Base Analysis Software (v2018B) was used to collect imaging data from cells. BD FACSDiva software (v 8.0.3) was used to collect flow cytometry data.
Data analysis	FlowJo (ver 10.6) used to analyze flow cytometry data. Prism (ver 8) used for all statistical analyses. R (ver 3.4) used to analyze gene expression data. living image (v 2.5) was used to analyze IVIS spectrum bioluminescence images.

For manuscripts utilizing custom algorithms or software that are central to the research but not yet described in published literature, software must be made available to editors and reviewers. We strongly encourage code deposition in a community repository (e.g. GitHub). See the Nature Research guidelines for submitting code & software for further information.

Data

Policy information about availability of data

All manuscripts must include a data availability statement. This statement should provide the following information, where applicable:

- Accession codes, unique identifiers, or web links for publicly available datasets
- A list of figures that have associated raw data
- A description of any restrictions on data availability

Raw RNAseq data supporting the findings of this study have been deposited in the National Center for Biotechnology Information Gene Expression Omnibus (NCBI-GEO) under accession number GSE161942 at https://www.ncbi.nlm.nih.gov/geo/query/acc.cgi? acc= GSE161942.

GSEA analysis was performed using default parameter settings using published gene sets (MSigDB, https://www.gsea-msigdb.org/gsea/ msigdb).

the source data underlying graphs in Figs. 1–7 and Supplementary Figs. 1-12 has been provided as a source data file. All data generated and analyzed are available from the corresponding author upon reasonable request.

Field-specific reporting

Please select the one below that is the best fit for your research. If you are not sure, read the appropriate sections before making your selection.

☒ Life sciences ☐ Behavioural & social sciences ☐ Ecological, evolutionary & environmental sciences

For a reference copy of the document with all sections, see nature.com/documents/nr-reporting-summary-flat.pdf

Life sciences study design

All studies must disclose on these points even when the disclosure is negative.

Sample size	The effect size is extremely high in CAR T cell experiments. In our studies, 10 mice per group would yield at least 90% power to detect a 2-fold difference in median survival based on a two-sided t-test with 5% Type I error. However, the effect sizes in CAR T cell experiments are large with animals either surviving or succumbing to the tumor; thus minimum of 4-6 animals is sufficient for these studies and was used in this manuscript.
Data exclusions	No data were excluded in our analyses.
Replication	all experiments were successfully replicated.
Randomization	Tumor bearing animals were randomized between treatment arms prior to initiation of therapy bases on the size of the tumor o ensure equal distribution of tumor sizes within each group. all other groups were randomly assigned.
Blinding	The experimenter injecting T cells and imaging did not know the grouping of animals at the time of the injection. Data were collected and analyzed at a later time point. therapeutic impact of CAR-T cells on tumor bearing mice is prominent leaving little to interpretation by the observer. We believe blinding would have not impacted out study results.

Reporting for specific materials, systems and methods

We require information from authors about some types of materials, experimental systems and methods used in many studies. Here, indicate whether each material, system or method listed is relevant to your study. If you are not sure if a list item applies to your research, read the appropriate section before selecting a response.

Materials & experimental systems

n/a	Involved in the study	
☐	☒	Antibodies
☐	☒	Eukaryotic cell lines
☒	☐	Palaeontology and archaeology
☐	☒	Animals and other organisms
☒	☐	Human research participants
☒	☐	Clinical data
☒	☐	Dual use research of concern

Methods

n/a	Involved in the study	
☒	☐	ChIP-seq
☐	☒	Flow cytometry
☒	☐	MRI-based neuroimaging

Antibodies

Antibodies used

flow cytometry: all surface antibodies were used at 1:200, except for CD3, CD4, CD8 (1:400); all intracellular antibodies were used at 1:200.
Anti-human:
PE anti-human IL-2 Biolegend cat#500306
PE anti-human IFN-γ Biolegend cat#502508
PE anti-human TNF-α Biolegend cat#502908
FITC anti-human CD3 Biolegend cat#317305
Brilliant Violet 421™ anti-human CD45 Biolegend cat#368521
PE anti-human CD107a (LAMP-1) Biolegend cat#328607
PE anti-human CD279 (PD-1) Biolegend cat#329905
PE anti-human CD223 (LAG-3) Biolegend cat#369305
PE anti-human CD25 Biolegend cat#302605
PE anti-human CD27 Biolegend cat#356405
PE anti-human CD19 Biolegend cat#392505
PE anti-human CD69 Biolegend cat#310905
PE anti-human Ganglioside GD2 Biolegend cat#357303
PE anti-human CD276 (B7-H3) Biolegend cat#331605

Anti mouse:
anti-mouse CD3ε Biolegend cat#100301
anti-mouse CD45 Biolegend cat#103133
anti-mouse CD107a Biolegend cat#121611
anti-mouse INFg Biolegend cat#505807
AffiniPure F(ab')$_2$ Fragment Goat Anti-Mouse IgG, F(ab')$_2$ fragment specific jackson immuno cat#115-006-072
AffiniPure F(ab')$_2$ Fragment Goat Anti-Mouse IgG, F(ab')$_2$ fragment specific-APC jackson immuno cat#115-006-072
AffiniPure F(ab')$_2$ Fragment Goat Anti-Mouse IgG, F(ab')$_2$ fragment specific-PE jackson immuno cat#115-006-072

Immunohistochemistry:
anti human CD3e Monoclonal eBioscience cat#14-0031-82
anti human PHOX2B Polyclonal eBioscience cat#PA5-35044
Anti mouse CD3 Monoclonal eBioscience Cat #MA5-14524

Validation

all primary anti-mouse antibodies were validated for flow cytometry by the manufacturers.

Anti-human:
PE anti-human IL-2 Biolegend cat#500306 https://www.biolegend.com/en-us/products/pe-anti-human-il-2-antibody-1351?GroupID=GROUP24
PE anti-human IFN-γ Biolegend cat#502508 https://www.biolegend.com/en-us/products/pe-anti-human-ifn-gamma-antibody-1011?GroupID=GROUP24
PE anti-human TNF-α Biolegend cat#502908 https://www.biolegend.com/en-us/products/pe-anti-human-tnf-alpha-antibody-1346
FITC anti-human CD3 Biolegend cat#317305 https://www.biolegend.com/en-us/products/fitc-anti-human-cd3-antibody-3644?GroupID=GROUP28
Brilliant Violet 421™ anti-human CD45 Biolegend cat#368521 https://www.biolegend.com/en-us/products/brilliant-violet-421-anti-human-cd45-antibody-14686
PE anti-human CD107a (LAMP-1) Biolegend cat#328607 https://www.biolegend.com/en-us/products/pe-anti-human-cd107a-lamp-1-antibody-4967?GroupID=GROUP28
PE anti-human CD279 (PD-1) Biolegend cat#329905 https://www.biolegend.com/en-us/products/pe-anti-human-cd279-pd-1-antibody-4412?GroupID=GROUP28
PE anti-human CD223 (LAG-3) Biolegend cat#369305 https://www.biolegend.com/en-us/products/pe-anti-human-cd223-lag-3-antibody-13549
PE anti-human CD25 Biolegend cat#302605 https://www.biolegend.com/en-us/search-results/pe-anti-human-cd25-antibody-616?GroupID=GROUP28
PE anti-human CD27 Biolegend cat#356405 https://www.biolegend.com/en-us/products/pe-anti-human-cd27-antibody-8371
PE anti-human CD19 Biolegend cat#392505 https://www.biolegend.com/en-us/products/pe-anti-human-cd19-antibody-16209?GroupID=GROUP483
PE anti-human CD69 Biolegend cat#310905 https://www.biolegend.com/en-us/products/pe-anti-human-cd69-antibody-1672
PE anti-human Ganglioside GD2 Biolegend cat#357303 https://www.biolegend.com/en-us/products/pe-anti-human-ganglioside-gd2-antibody-8408
PE anti-human CD276 (B7-H3) Biolegend cat#331605 https://www.biolegend.com/fr-ch/products/pe-anti-human-cd276-b7-h3-antibody-5573

Anti mouse:
FITC anti-mouse CD3ε Biolegend cat#100305 https://www.biolegend.com/en-us/products/fitc-anti-mouse-cd3epsilon-antibody-23
Brilliant Violet 421™ anti-mouse CD45 Biolegend cat#103133 https://www.biolegend.com/en-us/products/brilliant-violet-421-anti-mouse-cd45-antibody-7253
PE anti-mouse CD107a Biolegend cat#121611 https://www.biolegend.com/en-us/products/pe-anti-mouse-cd107a-lamp-1-antibody-4605
PE anti-mouse INFg Biolegend cat#505807 https://www.biolegend.com/en-us/products/pe-anti-mouse-ifn-gamma-antibody-997
AffiniPure F(ab')$_2$ Fragment Goat Anti-Mouse IgG, F(ab')$_2$ fragment specific jackson immuno cat#115-006-072 https://www.jacksonimmuno.com/catalog/products/115-006-072
AffiniPure F(ab')$_2$ Fragment Goat Anti-Mouse IgG, F(ab')$_2$ fragment specific-APC jackson immuno cat#115-006-072 https://www.jacksonimmuno.com/catalog/products/115-136-072
AffiniPure F(ab')$_2$ Fragment Goat Anti-Mouse IgG, F(ab')$_2$ fragment specific-PE jackson immuno cat#115-006-072 https://www.jacksonimmuno.com/catalog/products/115-136-072

Immunohistochemistry:
anti human CD3e Monoclonal eBioscience cat#14-0031-82 https://www.thermofisher.com/antibody/product/CD3e-Antibody-clone-145-2C11-Monoclonal/14-0031-82
anti human PHOX2B Polyclonal eBioscience cat#PA5-35044 https://www.thermofisher.com/antibody/product/PHOX2B-Antibody-

Polyclonal/PA5-35044
Anti mouse CD3 Monoclonal eBioscience Cat #MA5-14524 https://www.thermofisher.com/antibody/product/CD3e-Antibody-clone-SP7-Monoclonal/MA5-14524

Eukaryotic cell lines

Policy information about cell lines

Cell line source(s)	NB9464D is derived from murine TH-MYCN mice backcrossed to C57/B6J and transduced with murine GD2-GD3 synthetase. 282, 844 murine NBL cell lines were derived from TH-MYCN 129/SvJ mice. CHLA136, CHLA255, SKN-(BE)2, LAN5, LAN6 human NBL cell lines were derived from patients with progressive disease. Human cell lines were either established at CHLA or obtained from the Children's Oncology Group (COG) Cell Culture and Xenograft Repository (www.COGcell.org). GD2 expressing LAN6 (LAN6GD2+) cell lines were constructed by transducing wildtype LAN6 cells with GD2 synthase (B4GALNT1) and GD3 synthase (ST8SIA1). GFP and Luciferase positive cell lines were generated for in vitro and in vivo experiments using lentiviral expression. 293 T, jurkat and Raji and Eco-phoenix are purchased from ATCC.
Authentication	We validate each cell line every 6 months by frequent STR mapping
Mycoplasma contamination	Cells lines tested negative for mycoplasma contamination prior to sample generation. Samples were confirmed negative using MycoAlert (Lonza) Mycoplasma Detection Kit.
Commonly misidentified lines (See ICLAC register)	No commonly misidentified cell lines were used.

Animals and other organisms

Policy information about studies involving animals; ARRIVE guidelines recommended for reporting animal research

Laboratory animals	Mice used are C57B6 and NSG (NOD.Cg-Prkdc-scid /ILrg-tm1Wjl/SzJ, Jackson Laboratory) strains; males and females are equally used at 4-6 weeks of age for each experiment. Animals were housed in the Unit for animal care facility at the Children's Hospital of Los Angeles in compliance with the Institutional Animal Care and Use Committee regulations. Housing conditions at Children Hospital of Los Angeles: Mice were maintained in a specific pathogen free unit on a 13hr light: 11hr dark cycle. The animal rooms are provided with 100% fresh, HEPA filtered air at 10-15 air changes per hour. Room temperatures are controlled by a dedicated system within each room, and are maintained within the range of 72°F ± 2° F. Humidity levels are also controlled by a dedicated system within each room, maintained between 30-70% RH.
Wild animals	no wild animals were used in this study
Field-collected samples	no field-collected samples were used in this study
Ethics oversight	the Institute Animal Care and Use Committee (IACUC) at Children's Hospital of Los Angeles

Note that full information on the approval of the study protocol must also be provided in the manuscript.

Flow Cytometry

Plots

Confirm that:

☒ The axis labels state the marker and fluorochrome used (e.g. CD4-FITC).

☒ The axis scales are clearly visible. Include numbers along axes only for bottom left plot of group (a 'group' is an analysis of identical markers).

☒ All plots are contour plots with outliers or pseudocolor plots.

☒ A numerical value for number of cells or percentage (with statistics) is provided.

Methodology

Sample preparation	Various in vitro and in vivo experiments with different sample preparation were used as stated in the manuscript.
Instrument	LSRII 3 laser BD flow cytometer
Software	BD FACSDiva software (v 8.0.3) was used to collect flow cytometry data. Flowjo (ver. 10.6) was used to analyze the data
Cell population abundance	No sorting was conducted
Gating strategy	Gating strategy is explained in the Methods section of the manuscript.

☒ Tick this box to confirm that a figure exemplifying the gating strategy is provided in the Supplementary Information.
